# Capsule Switching among C:2b:P1.2,5 Meningococcal Epidemic Strains after Mass Immunization Campaign, Spain

**DOI:** 10.3201/eid0812.020081

**Published:** 2002-12

**Authors:** Belén Alcalá, Luisa Arreaza, Celia Salcedo, María J. Uría, Laura De La Fuente, Julio A. Vázquez

**Affiliations:** * Centro Nacional de Microbiología–Instituto de Salud Carlos III, Madrid, Spain

**Keywords:** Capsular switching, meningococcal strains, immunization campaign

## Abstract

A mass immunization campaign for 18-month to 19-year-olds was undertaken in Spain in 1996–1997 because of an epidemic of serogroup C meningococcal disease associated with a C:2b:P1.2,5 strain belonging to the A4 lineage. Surveillance for the “capsule-switching” phenomenon producing B:2b:P1.2,5 isolates was undertaken. Of 2,975 meningococci characterized, B:2b:P1.2,5 and B:2b:P1.2 antigenic combinations were found in 18 isolates; 15 meningococci were defined as serogroup B belonging to the A4 lineage.

In the early 1990s, an increasing number of serogroup C meningococcal strains were observed in Spain (1). Besides a change in the predominant serogroup, an increase in the incidence of the meningococcal disease associated with a new variant of serogroup C (2) was detected. These strains were characterized as C:2b:P1.2,5. Their frequency in serogroup C meningococci in Spain increased from 4.6% in 1993 to 65% in 1996 (2). Meningococcal strains characterized as C:2b:P1.2,5 have been described in other countries (3), but they have not been associated with a similar epidemiologic change. However, C:2a:P1.2 isolates belonging to the ET15 lineage have been responsible for epidemic waves in the Czech Republic and Canada (4,5).

As a result of the increase in such isolates in Spain, a mass immunization campaign focused at 18-month to 19-year-olds was conducted with the polysaccharide A+C vaccine in most of the country in 1996–1997 (6). Three years later, a new C conjugate vaccine was licensed in Spain. This vaccine was routinely introduced in autumn 2000 because an increase in the incidence of serogroup C cases was again detected.

By contrast, B:2b meningococci, which were frequently isolated during a previous epidemic period in Spain (7), represented 1.9% of the serogroup B strains characterized in our laboratory from 1990 to 1992 (8); none of them showed P1.2,5 serosubtype antigenic combinations. From 1995 to autumn 2000 we characterized 18 meningococcal strains as B:2b (14 as B:2b:P1.2,5 and 4 as B:2b:P1.2 isolates). Recombinant strains expressing serogroup B or C have been previously described as result of capsule-switching genetic mechanism (9,10). The aim of our study was to characterize those new B:2b meningococci variants. We used molecular typing methods (pulsed-field gel electrophoresis [PFGE] and multilocus sequence typing [MLST]) to determine the relationships among B:2b with the parental C:2b:P1.2,5 epidemic strain before and after the immunization campaign with the A+C polysaccharide vaccine.

## The Study

The Spanish Reference Laboratory for Meningococci routinely receives meningococci isolated from sterile sites for serogrouping, serotyping, and serosubtyping. From January 1995 to November 2000 (just before the new C conjugate vaccine was routinely introduced), the laboratory received 2,975 meningococcal strains to be characterized by serotyping and serosubtyping with monoclonal antibodies (8). The B:2b:P1.2,5 and B:2b:P1.2 antigenic combinations were found in 18 isolates (Table 1). All these strains were suspected of belonging to the A4 lineage and were fully characterized by PFGE and MLST as described previously (2,11); results were compared with those obtained among the C:2b:P1.2,5 epidemic strains. Two additional strains characterized as B:4:P1.2,5 were also included to determine if these antigenic combinations might be caused by similar genetics events.

**Table 1:** Distribution and characteristics of B:2b meningococcal strains with different antigenic combinations, Spain

Year	Identification no.	Antigenic expression	Pulse type	Clonal lineage by MLST^a^
1995	9813	B:2b:P1.2,5	NR^b^	ST1380^c^
	9976	B:2b:P1.2,5	PT7	ST8 (A4)
	10034	B:2b:P1.2,5	NR	ST1489^c^
1996	10317	B:2b:P1.2,5	PT7	ST8 (A4)
1997	11261	B:2b:P1.2	PT1	ST8 (A4)
	11327	B:2b:P1.2,5	PT7	ST8 (A4)
1998	12344	B:2b:P1.2,5	PT7	ST8 (A4)
	12366	B:2b:P1.2,5	PT7	ST8 (A4)
1999	12531	B:2b:P1.2	PT8	ST8 (A4)
	12644	B:2b:P1.2,5	PT8	ST8 (A4)
	12647	B:2b:P1.2,5	PT8	ST8 (A4)
	12792	B:2b:P1.2,5	PT8	ST8 (A4)
	12367	B:2b:P1.2,5	PT8	ST8 (A4)
2000	13602	B:2b:P1.2	PT38	ST8 (A4)
	13818	B:2b:P1.2,5	PT7	ST8 (A4)
	13872	B:2b:P1.2,5	PT4	ST66 (A4)
	13903	B:2b:P1.2	NR	ST162^c^
	14078	B:2b:P1.2,5	PT4	ST66 (A4)

^a^MLST, multilocus sequence typing. ^b^NR, pulse types not related to those found in C:2b:P1.2,5 strains. ^c^Clonal lineage not defined.

## Conclusions

Fifteen (83.3%) meningococci showed sequence types identified as representative of the A4 clonal lineage; this lineage also represents the genotype of the C:2b:P1.2,5 epidemic strain. The proportions of isolates belonging to the A4 clonal lineage were 75% and 85%, respectively, in both B:2b:P1.2 and B:2b:P1.2,5 strains. Seven of these 15 meningococci characterized as serogroup B belonging to the A4 lineage were isolated from patients who had never been immunized with the A+C polysaccharide vaccine. Three group B strains that were suspected by antigenic characterization of belonging to the A4 complex showed nonrelated sequence types (Table 1).

In a different study (data not shown), most of the C:2b:P1.2,5 epidemic strains grouped in two closely related pattern profiles by PFGE: PT7 and PT8. Table 1 shows the PFGE pattern profiles of the B:2b:P1.2,5 strains. Most of these isolates also showed the PT7 or the PT8 profile. Some strains showed minor pattern profiles already present among C:2b:P1.2,5 isolates (PT1, PT4, and PT38, all of them closely related to PT7 and PT8). Those strains showing PFGE pattern profiles that we did not find among the C:2b:P1.2,5 epidemic strain belonged to lineages different than A4 (Table 1). On the other hand, the B:4:P1.2,5 strains showed the same sequence type, ST33, associated with ET5.

 The frequency of the C:2b:P1.2,5 and B:2b:P1.2,5 meningococci of the A4 lineage is shown in Table 2. A slight increase of that group B isolates was found after the immunization campaign.

**Table 2:** Frequency of the B:2b:P1.2,5 and C:2b:P1.2,5 meningococcal strains characterized by the laboratory, 1995–2000

Year	No. of strains received by the laboratory	No. of C:2b:P1.2,5 (%)	No. of B:2b:P1.2,5 (%)
1995	254	56 (22%)	1 (0.4%)
1996	380	176 (46.3%)	1 (0.26%)
1997	687	313 (45.5%)	2 (0.3%)
1998	554	149 (26.9%)	2 (0.36%)
1999	598	144 (24.1%)	5 (0.84%)
2000	502	97 (19.3%)	4 (0.8%)

Recombinant strains expressing serogroup B or C have been previously described as resulting from a capsule-switching genetic mechanism (9,10). A similar event with W135 isolates has been recently described (12). Thus, all the serogroups should be capable of changing to any other. However, the relevance of this phenomenon has not been fully described. In our surveillance analysis, the group B strains of the A4 lineage appeared before the vaccination campaigns; this finding differs from results of an analysis conducted in Canada after a similar surveillance (10). Our findings show that those genetic variants are being produced in the meningococcal population at random and that a variant’s appearance is not necessarily related to mass immunization campaigns. In fact, these group B meningococci belonging to the A4 lineage were also isolated in some of the regions that used the vaccine on a small scale (13).

However, the increased number of these B:2b:P1.2,5 strains from the A4 lineage during the study period might indicate a positive selection caused by mass immunization campaigns during 1996 and 1997 (Table 2). Seven (50%) of these strains were isolated from patients who did not receive A+C vaccine, indicating that the individual immune status should not be a critical factor for developing meningococcal disease with these serogroup B strains of the A4 lineage rather than other clonal lineages. Nasopharingeal competition might be an important factor in the spread of these group B strains, as has been suggested to explain the spread of serogroup B meningococci belonging to the ET15 lineage (10). Theoretically, however, the C:2b strains of the A4 lineage and the B:2b meningococci also belonging to the A4 lineage should have a very similar genetic background with the exception of a locus in the capsular operon (9). In fact, both types of strains showed not only the same or similar sequence type by MLST but also a very similar genetic profile by PFGE (data not shown). Thus, a similar epidemic potential in these two capsular variants might be expected. Nevertheless, those strains with a group C polysaccharide capsule maintained a major epidemic even after a mass immunization campaign (Table 2) and even when these group C strains were not common in the carrier population (14).

Whether a similar observation can be made for the serogroup B strains belonging to the A4 clonal lineage is not clear; the small increase in these serogroup B strains does not appear to be linked with increased epidemic potential. Thus, the capsular polysaccharide appears to be the only differing factor between these two types of meningococci. Once again, the high number of serogroup B strains in asymptomatic carrier population (14) associated with a natural immunity, might partially explain the different epidemic potential of the two genotypes. However, the nature of the group C polysaccharide alone does not explain why C:2b:P1.2,5 strains were responsible for an important epidemic wave in Spain in 1996–1997. Some other factors, such as the amount of polysaccharide, might explain differences in virulence (15).

Two meningococci characterized as B:4:P1.2,5 were included to analyze if some other antigenic combinations might appear as result of different genetic events. This possibility was not confirmed in our study but should be more accurately analyzed in the future.

In 2000, a new increase in cases of group C meningococcal disease was detected in some regions of Spain (6). Because of these data, Spanish health authorities made the decision to include a new group C conjugate vaccine in the routine infant immunization schedule beginning in autumn 2000. How the two different vaccines against group C meningococci (polysaccharide and conjugate) influence the selection of serogroup B belonging to the A4 lineage strains in Spain that have had a capsular-switching event would be an interesting future topic of research.
